# Pseudotumor Cerebri and Hemolytic Uremic Syndrome, A Rare Association

**DOI:** 10.1002/ccr3.71526

**Published:** 2025-11-24

**Authors:** Aasim Ali, Armaghana Abdullah, Mukesh Kumar Sharma, Anousha Tanveer, Muhammad Abdullah Mushtaq, Muhammad Mujtaba, Summaya Mushtaq

**Affiliations:** ^1^ Allied Hospital Faisalabad Sargodha Road Faisalabad Faisalabad Punjab Pakistan; ^2^ Dr. Dhankuta District Hospital Dhankuta NP Nepal; ^3^ Shalamar Medical and Dental College Lahore Lahore Punjab Pakistan

**Keywords:** acetazolamide, hemolytic uremic syndrome, papilledema, pseudotumor cerebri, sixth nerve palsy

## Abstract

Hemolytic uremic syndrome can present with central nervous system involvement. Idiopathic intracranial hypertension (IIH) is among the possible neurological manifestations of HUS, and clinicians should remain vigilant for extrarenal complications while treating patients with HUS.

## 1 Introduction

Idiopathic intracranial hypertension (IIH), formerly known as pseudotumor cerebri, is not an uncommon condition, having an incidence of 0.9–1.56 per 100,000, which usually presents with headache, diplopia, tinnitus, vomiting, and blurring of vision. It is more common in individuals aged 12–25 years [1]. Its diagnosis is usually established after excluding other possible causes of raised intracranial pressure, including any space‐occupying lesion or sinus thrombosis. Raised intracranial pressure usually ranges above 25 cm CSF (H2O), and few radiological and clinical examination findings suggest IIH.

To date, no specific cause for IIH has been established. But its association with various conditions has been reported, such as SLE [2], idiopathic thrombocytopenic purpura, obesity, polycystic ovarian syndrome, adrenal insufficiency, Cushing's disease, hyperparathyroidism, primary hypothyroidism [3], tetracycline and OCPs, hypervitaminosis A, chronic renal failure, and anemia [4].

In this case report, we present a rare case of association between IIH and hemolytic uremic syndrome (HUS).

## 2 Case History/Examination

An Asian female in her twenties with a previous BMI of 25 kg/m^2^ presented in the emergency department of our hospital with an unremarkable past medical history and complaints of anuria and body swelling for 2 days following multiple episodes of watery, nonbloody loose stools 5 days ago. She neither mentioned any specific food that she had taken before the loose stools nor had any medication for them. She was alert and had mild dyspnea while lying down.

On examination, she had pitting edema in the lower limbs and on the sacrum, mild conjunctival pallor, leukonychia, with a respiratory rate of 26 breaths/m, HR of 105 beats/m, oxygen saturation of 92%, blood pressure of 140/90 mmHg, and temperature was normal. She had mild periorbital puffiness. There were slightly decreased breath sounds at the base of the back of the lungs and an S3 gallop on cardiac auscultation. Neurological and abdominal examinations were unremarkable on day one of admission. She had just 100 mL (normal more than 500 mL/24 h) of urine output in the last 24 h.

## 3 Differential Diagnosis, Investigations, and Treatment

Complete metabolic profile showed severely deranged kidney functions including raised levels of urea, creatinine, and uric acid in blood; elevated leukocytes (23,800/mm^3^) with low platelet count (36,000/mm^3^, normal 150,000–300,0003/mm^3^) and hemoglobin; increased reticulocytes (3.5%, normal: 1%–2%); along with lactate dehydrogenase (2062 U/L, normal 140–280 U/L); mounted fibrin degradation products (1600 ng/mL, normal less than 200 ng/mL); and schistocytes on peripheral blood picture all suggesting cellular breakdown and thrombus formation in blood vessels as seen in microangiopathic hemolytic anemia. Her prothrombin time and activated partial thromboplastin time were normal. Further testing, such as cANCA, pANCA, ANA levels, anti‐SSA, anti‐SSB antibodies, and ASO titers, was performed for alternative diagnoses, but the results were unremarkable. Her ADAMTS13 activity was also within normal range. Shiga toxin (Stx) was detected in stool, and a diagnosis of HUS was established. All laboratory findings are mentioned in Table 1.

**TABLE 1:** Patient's investigations and laboratory findings over the clinical course of her illness.

Test name	Day 1	Day 2	Day 4	Day 6	Day 8	Day 11	Day 13	References/Units
Blood urea	126	171		173	160	77	40	10–15 mg/dL
Blood urea nitrogen	58.9	79.9		80.8	74.8	36.1	18.6	6–23 mg/dL
Creatinine (Serum)	7.47	9.84		9.88	11.2	4.51	2.33	0.55–1.02 mg/dL
eGFR	7	5		5	4	13	27	> 60 mL/min/1.73m^2^
WBCs	23,800	23,300	35,100	59,200	14,700	9000	8300	4000–11,000/mm^3^
Platelets	34,000	35,000	124,000	46,000	178,000	518,000	386,000	140,000–425,000/mm^3^
Schistocytes and NRBCs	+	+	+	+	+			Normally absent
Sodium/Potassium	141/5.1		146/4.9		142/3.8	146/4.3		135–145/3.5–5.5 meq/L
Lactate dehydrogenase		2062				312		135–214 U/L
Fibrin degradation products		6500 mg/L				180		< 200 mg/L

Two sessions of hemodialysis per week for deranged renal functions, along with adequate maintenance of fluid and electrolyte balance, and sessions of plasmapheresis were started. Broad‐spectrum antibiotics were added to her treatment regimen. One pint of packed cell volume and one Mega Unit (platelet concentrate) were transfused when her hemoglobin fell to 7.3 and platelets to 26,000/μl, respectively.

**FIGURE 1:**
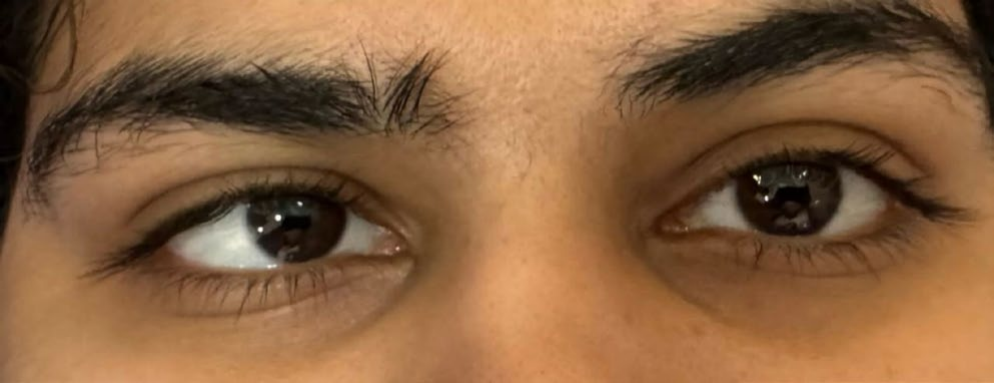
Convergent strabismus. Patient has medial deviation of the right eye because of paresis of the right sixth nerve palsy.

On the sixth day of admission, patients started complaining about blurring of vision in eyes, diplopia, bilateral tinnitus, headache, which worsened while coughing and sneezing, and convergent strabismus as noted by her mother; shown in Figure 1.

**FIGURE 2:**
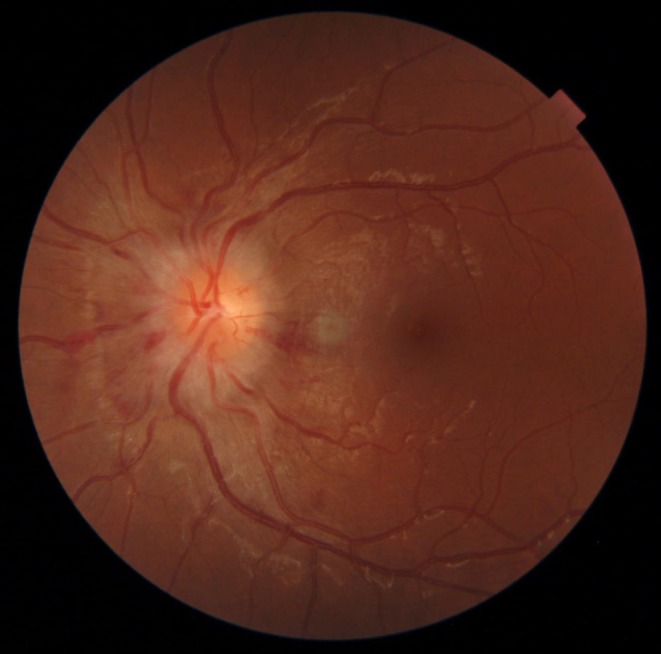
Grade IV papilledema. Elevation of the entire nerve head, obscuration of all borders, peripapillary halo, and total obscuration on the disc of a segment of a major blood vessel.

**FIGURE 3:**
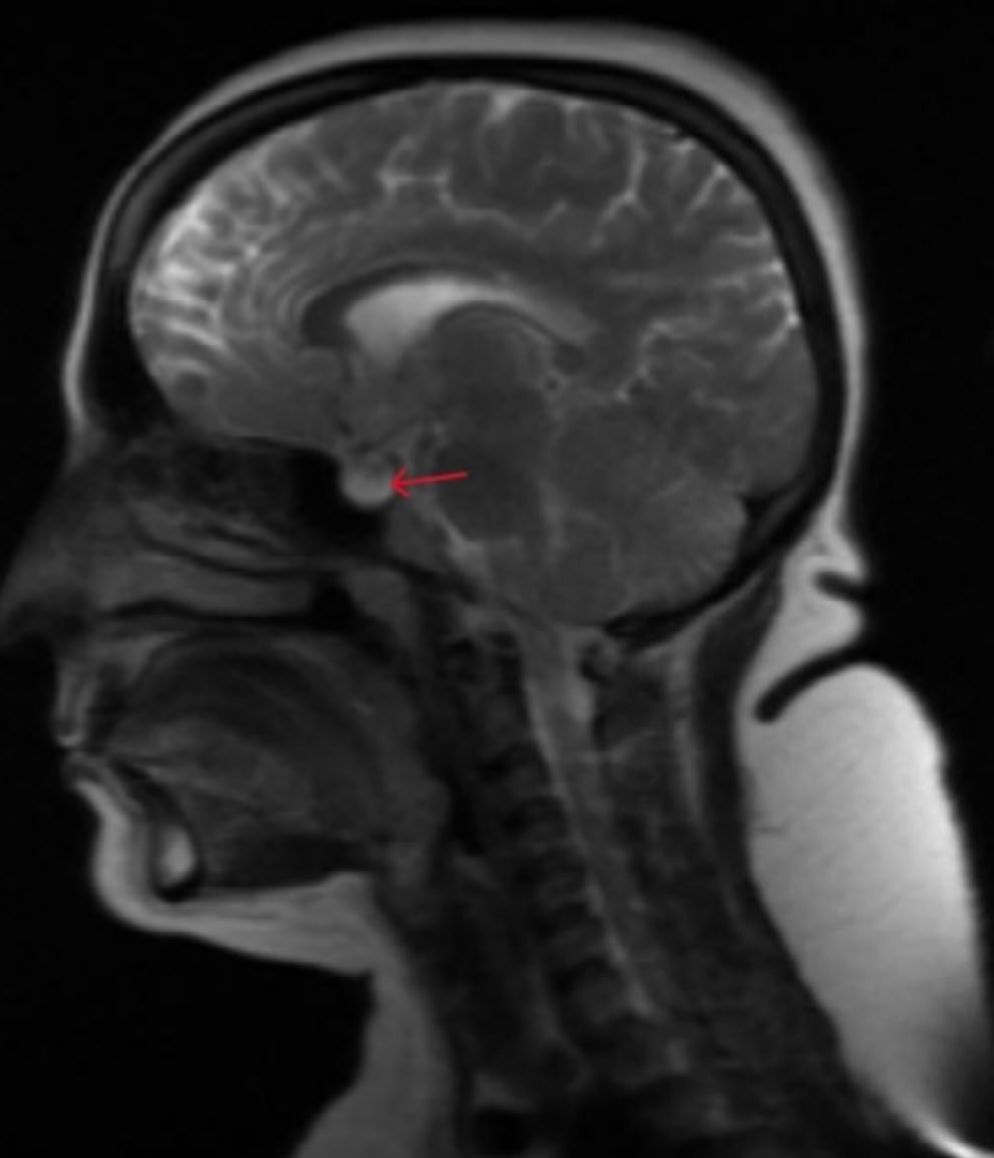
MRI brain sagittal view T2 sequence shows empty sella as indicated by the red arrow.

Clinical examination revealed right‐sided sixth nerve palsy, enlarged blind spot, and mildly decreased visual acuity, along with grade IV papilledema as shown in Figure 2. Her cerebrospinal fluid opening pressure in the supine position with straight legs was elevated to 300 mm of H2O (less than 250 mm of H2O), but cerebrospinal fluid complete microscopic examination was normal, as shown in Table 1. Magnetic resonance imaging revealed empty sella along with straightening of the posterior sclera, shown in Figure 3, suggesting Pseudotumor cerebri as no secondary cause, including venous sinus thrombosis, space‐occupying lesion, etc., of raised intracranial pressure was found on neuroimaging. Pseudotumor cerebri diagnosis was established using modified dandy criteria.

For IIH, we added acetazolamide in a renal adjusted dose, that is, 250 mg twice a day, as she was on hemodialysis along with furosemide 80 mg twice a day, a higher dose keeping deranged kidney functions. Sessions of hemodialysis were also continued. Five sessions of plasmapheresis were completed.

## 4 Conclusion and Results

Frequent fundoscopic examinations and electrolyte monitoring were done to assess the evolution of papilledema and to see any electrolyte or acid‐base abnormality possibly caused by acetazolamide or furosemide. Her visual acuity and strabismus improved over a period of one and a half weeks, and her last reported eGFR (estimated glomerular filtration rate) was 53 mL/min/1.73m^2^, and creatinine was 1.3 mg/dL. Her renal replacement therapy was discontinued.

## 5 Discussion

HUS was first described by Gasser and colleagues in 1955. It is defined by the triad of microangiopathic hemolytic anemia, thrombocytopenia, and acute renal failure. The differential diagnosis includes sepsis and disseminated intravascular coagulation (DIC), SLE, and TTP. Of note, in both HUS and TTP, other markers of coagulation, including PT and aPTT, are normal, thus distinguishing these from DIC. HUS is estimated to occur with a frequency of 0.3–3.3 per 100,000 children worldwide, although it is noted that the incidence has been increasing in the past decade; it is usually diagnosed by assessing Shiga toxin in the stool of infected patients. Typically, it is caused by Shiga toxin (Stx) producing *Escherichia coli* , and in atypical cases, it may be caused by some autoimmune processes, complement deficiencies, or by infectious agents [1]. In our patient, raised levels of lactate dehydrogenase and fibrin degradation products, low platelet count, deranged renal function tests, normal PT, aPTT, ADAMTS13 activity, and detection of Shiga toxin in stool helped in establishing the diagnosis of typical HUS.

The two principal target organs of Stx‐mediated damage are the kidney and the brain. The reported rate of brain involvement in patients with HUS widely varies between 10% and 52% due to the unstrict definition of neurologic involvement among reported studies. In a recent study that involved a retrospective analysis of 202 children aged < 16 years with confirmed STEC‐caused HUS from 2005 to 2018, the authors reported a rate of 11% based on defining neurologic involvement by seizures, encephalopathy, or focal neurologic deficit, and considering features such as irritability or lethargy to be nonspecific [5]. Most atypical cases of HUS are associated with central nervous system involvement, but in our case, IIH was associated with typical Shiga toxin‐mediated HUS.

Central nervous system manifestations may include seizures, stupor, coma, ataxia, chorea, dystonia, or hemiparesis, and headache [6], but in our case, HUS was associated with IIH, characterized by isolated increased intracranial pressure (ICP) of unknown cause. By definition, intracranial disorders such as a meningeal process or cerebral venous thrombosis must be ruled out to make a diagnosis of IIH [7]. We established the diagnosis of PTC by modified Dandy criteria [7], as our patient had headache, blurring of vision, papilledema, right‐sided sixth nerve palsy, CSF pressure was elevated, but complete examination of CSF was normal, and MRI did not show any alternate diagnosis for raised intracranial pressure. While MRI findings in IIH are often unremarkable, our patient exhibited empty sella—a classic radiographic feature of intracranial hypertension [6]. This finding further supports the diagnosis of IIH in this clinical context.

To date, no specific cause for IIH has been established. But its association with various conditions has been reported, such as idiopathic thrombocytopenic purpura, SLE [2], obesity, polycystic ovarian syndrome, adrenal insufficiency, Cushing's disease, hyperparathyroidism, primary hypothyroidism [3], Tetracycline and OCPs, hypervitaminosis A, anemia [4], and chronic renal failure. Our patient was previously healthy and was not suffering from any of the above‐mentioned conditions. She did not complain about such headaches and vision issues in the past. This all depicts that the patient developed this IIH in association with HUS, as no alternative cause was found. To the best of our knowledge, no prior case report has documented such an association.

The pathogenesis of central nervous system involvement in HUS parallels renal involvement. Shiga toxin in typical HUS and alternative complement pathway dysregulation in atypical HUS lead to uncontrolled complement activity, endothelial injury, and thrombotic microangiopathy (TMA), causing multi‐organ damage [8]. A speculative but plausible mechanism is that endothelial injury and complement‐mediated microthrombosis within cerebral venules could impair CSF absorption and venous drainage, thereby contributing to raised ICP. Similar mechanisms have been proposed in systemic lupus erythematosus (SLE)–associated IIH, suggesting a potential shared pathogenic pathway [2, 7]. Recent literature further emphasizes complement dysregulation and neuroinflammatory changes as critical drivers of IIH pathophysiology [8, 9], lending indirect support to our observation.

HUS is typically treated with supportive care, including fluid balance and hemodialysis. In our case, we performed five sessions of plasmapheresis in addition to hemodialysis, fluid and electrolyte maintenance, and transfusion of platelets and RBCs. According to the American Society of Apheresis, plasmapheresis is a Category III recommendation with neurologic involvement of STEC‐HUS and a Category IV recommendation in STEC‐HUS without neurologic involvement [10]. Acetazolamide and furosemide were also initiated to manage papilledema and prevent vision loss. Therapy continued for 2 weeks, leading to significant improvement in headache, visual symptoms, and papilledema. Neurological complications are managed with condition‐specific therapies, and in this case, IIH was managed with acetazolamide, furosemide, and plasmapheresis.

In conclusion, HUS can manifest with extra‐renal complications, particularly neurological involvement. Among these, IIH represents a recognized but underappreciated presentation. Management extends beyond hemodialysis to include plasmapheresis and renal‐adjusted acetazolamide therapy when CNS symptoms emerge. Clinicians should maintain a high index of suspicion for this broad clinical spectrum when evaluating HUS patients, as timely recognition of neurological manifestations is critical for optimal management.

## Author Contributions

**Aasim Ali:** writing – original draft. **Armaghana Abdullah:** supervision. **Mukesh Kumar Sharma:** data curation. **Anousha Tanveer:** writing – review and editing. **Muhammad Abdullah Mushtaq:** software. **Muhammad Mujtaba:** writing – review and editing. **Summaya Mushtaq:** validation.

## Funding

The authors received no specific funding for this work.

## Consent

Written and informed consent was obtained from the patient to publish her clinical health‐related information.

## Conflicts of Interest

The authors declare no conflicts of interest.

## Data Availability

Data sharing not applicable to this article as no datasets were generated or analysed during the current study.
